# Signaling mechanisms governing the environmental modulation of fruit quality development

**DOI:** 10.1093/hr/uhag005

**Published:** 2026-01-07

**Authors:** Changsheng Zhai, Yating Li, Jie Li, Pingyin Guan, Juan Jin, Wensuo Jia

**Affiliations:** College of Horticulture, China Agricultural University, Beijing 100193, China; College of Horticulture, China Agricultural University, Beijing 100193, China; College of Horticulture, China Agricultural University, Beijing 100193, China; College of Horticulture, China Agricultural University, Beijing 100193, China; Institute of Fruits and Vegetables, Xinjiang Academy of Agricultural Sciences, Urumqi 830091, China; College of Horticulture, China Agricultural University, Beijing 100193, China; Institute of Fruits and Vegetables, Xinjiang Academy of Agricultural Sciences, Urumqi 830091, China

## Abstract

The control of fruit quality is of major scientific, nutritional, and commercial importance. In addition to being influenced by the intrinsic characteristics of each fruit species, fruit quality development is largely modulated by environmental factors. The environmental modulation of fruit quality primarily involves a signal transduction process that links environmental perception to the transcriptional or post-transcriptional regulation of key enzymes participating in fruit quality–associated metabolisms. Over the past decades, the effects of environmental factors on fruit quality traits have been extensively studied, and increasing attention has been directed toward elucidating the signaling mechanisms that govern this environmental modulation. However, knowledge in this research area has not yet been systematically summarized. In this review, we first provide an overview of the physiological and molecular bases underlying the modulation of fruit quality development by the three major environmental factors: water deficit, salinity, and temperature stresses. We then summarize recent advances in understanding the signaling mechanisms that mediate the environmental modulation of fruit quality development. Finally, we propose several perspectives to facilitate comprehension and guide future research endeavors.

## Introduction

Fleshy fruits constitute an important part of the human diet due to their rich nutritional value [1, 2]. Fruit quality is defined as the combination of characteristics that determine consumer acceptance, including color, nutritional content, taste, flavor, and texture [3]. It serves as the primary determinant of both the nutritional and commercial values of fruits. Therefore, the control of fruit quality has long been a major focus of horticultural producers and scientists [4, 5]. In addition to being influenced by the intrinsic characteristics of fruit species, fruit quality development is strongly affected by environmental factors such as light, temperature, water availability, soil salinity, and nutrient status. Severe stress caused by these factors can significantly reduce fruit yield and quality, whereas mild stress may improve fruit quality. Therefore, studies on the mechanisms underlying the environmental modulation of fruit quality development are of great significance for improving fruit quality control.

Living in an ever-changing environment, plants have evolved mechanisms to perceive and respond to environmental signals, allowing them to survive and thrive under adverse climatic conditions [6, 7]. These mechanisms rely on cellular signaling systems in which numerous signaling molecules form complex cascades that connect environmental signal perception to the regulation of cellular metabolism in response to abiotic stresses. Membrane proteins, particularly receptor-like protein kinases (RLKs), have increasingly been recognized as sensors of environmental signals [8, 9]. In addition, various protein kinases, such as sucrose nonfermenting 1–related kinases (SnRKs), calcium-dependent protein kinases (CDPKs), calcineurin B-like protein (CBL)-interacting protein kinases (CIPKs), and mitogen-activated protein kinases (MAPKs), have been well established as crucial intermediates that mediate the perception of environmental signals and their conversion into stress responses [10, 11]. A wide range of transcription factors (TFs) act as the downstream targets of these kinases, thereby regulating the expression of genes involved in stress-responsive metabolisms.

Over the past decades, the effects of environmental factors on fruit quality development have attracted increasing attention from both horticultural scientists and producers. It is well established that changes in environmental conditions may significantly influence nearly all aspects of fruit quality traits, including color, sugar–acid ratio, taste, flavor, antioxidant activity, and texture [12–15]. Importantly, growing evidence suggests that abiotic stress–associated signaling pathways are involved in the modulation of fruit quality development [16, 17], providing important insights into the molecular bases underlying fruit quality regulation. However, the signaling mechanisms governing the environmental modulation of fruit quality development have not yet been systematically summarized. In this review, we first present an overview of the physiological and molecular bases underlying fruit quality modulation—specifically, the physiological parameters related to fruit quality and the regulation of genes encoding TFs and key enzymes involved in quality-associated metabolisms—under different environmental conditions, with particular emphasis on water availability, soil salinity, and temperature. We then summarize recent advances in understanding the signaling mechanisms responsible for the environmental modulation of fruit quality development. Finally, we propose several prospective research directions to aid comprehension and guide future research endeavors.

## Physiological and molecular bases for the environmental modulation of fruit quality traits

Fruit quality traits encompass a wide range of physio-biochemical parameters, including fruit size, color, sugar content, acid content, taste, flavor, and texture. The development of these parameters is governed by cellular metabolic processes that are regulated by key enzymes in the corresponding metabolic pathways. Upstream of these key enzymes are diverse TFs, which together form signaling modules consisting of TFs and their target genes (i.e., the genes encoding the key enzymes involved in fruit quality development). To date, many such signaling modules have been identified as participants in the environmental modulation of fruit quality development. Therefore, the identification and characterization of TFs and key metabolic enzymes, particularly their signaling modules, are essential for elucidating the mechanisms by which fruit quality develops in response to environmental signals.

### Water deficit

#### Pigment metabolism

Anthocyanins, the primary pigments responsible for coloration in many fruits, are synthesized through the phenylpropanoid pathway. Many studies have demonstrated that water deficit strongly affects pigment metabolism. In jujube (*Ziziphus jujuba* Mill.), several genes encoding anthocyanin synthase (ANS) are induced by water deficit [18]. In wild tomato (*Solanum peruvianum*), water deficit induces anthocyanin biosynthesis via the upregulation of *SpAN2*, which encodes an MYB-type TF, and *SpDFR*, which encodes a key enzyme in the late flavonoid biosynthetic pathway. However, whether SpAN2 directly targets *SpDFR* has not yet been demonstrated [19]. In grapevine, moderate water deficit promotes anthocyanin accumulation in red grape skins. The microRNA *vv-miR156b* is induced by water deficit and is regulated by VvAREB2, an abscisic acid (ABA)-responsive TF that can directly bind to the *Vv-miR156b* promoter. Manipulation of *VvSBP8* and *VvSBP13*, two *miR156B* targets that encode transcriptional repressors, modulates anthocyanin accumulation. Furthermore, VvSBP8 and VvSBP13 interact with VvMYC1 and VvMYBA1, resulting in the repression of anthocyanin biosynthesis [20]. In apple (*Malus domestica*), manipulation of *MdERF38* expression influences anthocyanin accumulation, indicating that the ERF TF plays a crucial regulatory role in anthocyanin biosynthesis. Moreover, MdERF38 interacts with MdMYB1, a positive modulator of anthocyanin biosynthesis. The transcripts of *MdERF38*, *MdMYB1*, and two key enzymatic genes in the anthocyanin biosynthesis pathway—*MdDFR* and *MdUF3GT*—are strongly induced by drought stress, suggesting that the MdERF38–MdMYB1 module modulates drought-induced anthocyanin biosynthesis [21].

#### Sugar metabolism

Sugar content and composition are major indicators of fruit quality [14]. Many studies have indicated that soil water availability strongly influences fruit sugar content. For example, in peach (*Prunus persica* L*.*), severe water deficit increases glucose, fructose, and sorbitol concentrations in the flesh by 12% to 70% [14, 22, 23]. In apple, water deficit increases sucrose content by 27% [24], whereas in tomato (*S. lycopersicum* ‘Micro Tom’), moderate water deficit significantly elevates glucose and fructose concentrations by 86.70% and 19.83%, respectively [25].

Sugar accumulation is closely related to the expression of genes encoding enzymes involved in sucrose metabolism, such as invertase and sucrose synthase. In citrus (*Citrus reticulata* Blanco), moderate water stress significantly decreases the transcript levels of *CitSUS1*, *CitSUS3*, *CitSUS4*, and *CitSUS5*, whereas *CitSUS2* expression increases by 2.5-fold compared to the control [26]. Transcript levels of cell wall invertase genes (*CwINV2* and *CwINV6*) and sucrose synthase genes (*SUS2* and *SUS6*) are significantly increased in response to water deficit, promoting soluble sugar accumulation via enhanced expression of sink strength– and transport-related genes [27]. Additionally, vacuolar pyrophosphatase proton pumps (V-PPases) appear to play an important role in sucrose accumulation in citrus, as *CsVPP-1* and *CsVPP-2* are upregulated under drought conditions, correlating with increased sucrose content [28].

#### Organic acid metabolism

In addition to sugar content, the levels of organic acids and the sugar/acid ratio are important determinants of fruit quality. In *Vitis vinifera* ‘Cabernet Sauvignon’, water deficit increases malic and tartaric acid contents by 23.9% and 16.6%, respectively, compared with those observed under well-watered conditions [29]. In *V. vinifera* ‘Ugni blanc’, tartaric acid content in ripe fruits is considerably higher under hotter and drier climates than under cooler and wetter conditions, indicating that air humidity influences organic acid accumulation [30]. In tomato, severe water deficit significantly increases citric and quinic acid concentrations throughout fruit development [22, 31]. Meanwhile, mild water deficit increases proline, aspartic acid, malic acid, citric acid, and ascorbic acid concentrations, raising total amino and organic acid contents by 18.91% and 26.12%, respectively [32]. In grapefruit (*C. paradisi* Mac.), severe water deficit increases acidity and reduces sugar concentrations, whereas moderate water stress elevates flavonoid and phenolic contents but decreases lycopene levels [33]. In citrus, citric acid accumulation has been associated with ACC oxidase (ACO) activity [34]: reduced *ACO* expression contributes to citrate accumulation during ripening. Similarly, in tomato, inhibition of *SlACO3a* and *SlACO3b* expression increases citrate content [35]. In the apple cultivar ‘Yanfu 3’, overexpression of *MdPEPC4* (encoding phosphoenolpyruvate carboxykinase) increases malate content in response to 4% PEG treatment [36].

#### Antioxidant metabolism

Antioxidant activity is an important fruit quality indicator due to its importance to human health [37, 38]. Fruit antioxidants, such as anthocyanins, flavonoids, carotenoids, and phenolic compounds, are major components of fruit pigments. Many studies have demonstrated that antioxidant metabolism in fruits is strongly affected by water availability. Because anthocyanins have been discussed earlier, this section focuses on other antioxidants, such as flavonoids and carotenoids. In grapevine, water deficit upregulates the expression of multiple antioxidant metabolism–related genes, such as *UFGT* (encoding UDP-glucose:flavonoid 3-O-glucosyltransferase), *CHS2* and *CHS3* (chalcone synthases), *F3H* (flavanone 3-hydroxylase), and *F3′5′H* (flavonoid 3*′*,5*′*-hydroxylase) [39]. In strawberry (*Fragaria × ananassa*), mild drought increases phenolic, anthocyanin, l-ascorbic acid contents, as well as overall antioxidant activity, while upregulating genes involved in antioxidant metabolism, such as *PAL* (encoding phenylalanine ammonia-lyase), *4CL* (ρ-coumarate ligase), *C4H2* (cinnamate 4-hydroxylase 2), *DFR* (dihydroflavonol 4-reductase), and *FLS* (flavonol synthase) [40]. In tomato, water deficit increases total phenolic content but reduces total carotenoid content [41]. Ewas *et al.* [42] demonstrated that SlMX1, a MIXTA-like MYB TF, promotes carotenoid accumulation and enhances drought resistance.

#### Aroma metabolism

The production of aroma and volatile compounds is a critical determinant of fruit quality. However, few studies have investigated the effects of water availability on fruit aroma production. In *V. vinifera* ‘Pinot Noir’, volatile compound content increases slightly in response to water deficit [43]. In strawberry, root inoculation with Antarctic fungi mitigates drought stress, and fruits from inoculated, drought-stressed plants show significant upregulation of *PDC* and *AAT* genes, leading to increased total volatile ester production [44].

### Salinity

#### Pigment metabolism

Several studies have investigated the effects of salinity stress on fruit pigmentation. Borghesi *et al.* [45] compared anthocyanin accumulation in three tomato genotypes (*Anthocyanin fruit type*, *Atroviolaceum*, and *Sun Black*) under salinity stress conditions. The results showed that salinity stress leads to a 2- to 3-fold increase in lycopene content. Saline treatment increases total anthocyanin accumulation by 2-fold in *Sun Black* fruits but reduces it 10-fold in *Anthocyanin* fruits, indicating that the effects of salinity stress on anthocyanin accumulation vary among cultivars. Li *et al.* [46] reported that moderate salinity (20 and 60 mM) increases anthocyanin and soluble solid contents in grape berries, whereas severe salinity stress (100 and 150 mM) decreases glucose and fructose contents, suggesting that the effects of salinity stress on sugar and anthocyanin metabolisms depend on stress severity. By contrast, a recent study by Yağcı *et al.* [47] showed that severe salinity stress (150 mM NaCl) markedly suppresses sugar metabolism, reducing glucose and fructose contents by approximately 85% and 82%, respectively, while increasing total anthocyanin accumulation by up to 60%. In apple, gibberellin 2-oxidase (GA2ox) has been reported to play a role in anthocyanin accumulation. The expression of *MdGA2ox7* is regulated by salinity stress, and its overexpression enhances anthocyanin biosynthesis in apple calli by activating genes involved in anthocyanin synthesis [48]. Li *et al.* [49] identified a UDP-glycosyltransferase–encoding gene in the apple genome, *MdUGT83L3*, which is strongly induced by salinity stress. Overexpression of *MdUGT83L3* increases anthocyanin accumulation in apple calli, implying its role in mediating salinity stress-induced anthocyanin biosynthesis. Similarly, *MdHsp18.2b*, which encodes a heat-shock protein, is significantly upregulated under salinity stress, and its overexpression enhances anthocyanin accumulation in apple calli [50].

#### Sugar and organic acid metabolisms

Studies examining the effects of salinity stress on sugar metabolism in tomato fruit revealed that the responses vary among cultivars. For example, salinity stress under 120 mM NaCl increases fructose and glucose contents in landraces but has no effect on hexose levels in ‘UC-82B’ [51]. Similarly, salinity stress significantly raises sucrose content in ‘Pimiento’ and ‘Muchamiel Aperado’ but not in ‘Moneymaker’ [52]. In grape berries, the effects of salinity stress on sugar metabolism vary with stress severity: moderate salinity (20 and 60 mM) increases glucose, fructose, and sucrose contents, whereas severe stress (100 and 150 mM) decreases glucose and fructose levels [46]. *SWEET* genes, which encode a novel class of sugar transporters [53], have been increasingly recognized as key regulators of salinity stress–induced modulation of fruit quality development [53–55]. In banana, jujube, and cranberry, 13–25 *SWEET* gene family members have been identified, many of which respond to salinity stress [53–55]. Invertase also plays a crucial role in sugar metabolism. In fig (*Ficus carica* L.), Mascellani *et al.* [56] reported that the transcripts of alkaline–neutral and acid invertases are upregulated in ripe fruits under salinity stress. However, genetic evidence confirming their roles in salinity stress-induced fruit quality modulation remains lacking.

Studies investigating the effects of salinity stress on organic acid metabolism in fruits are limited. Yin *et al.* [57] reported that during fruit development, most amino acids increase in response to salinity stress, whereas organic acids show minimal change. Throughout fruit development, phosphoenolpyruvate carboxylase 2 and phosphoenolpyruvate carboxykinase display contrasting expression patterns between early development and ripening stages, suggesting a shift in carbohydrate metabolism after the turning stage. Organic acid metabolism is closely associated with the tricarboxylic acid (TCA) cycle and the gamma-aminobutyric acid (GABA) pathway. Many genes involved in the TCA cycle respond to salinity stress, although the specific genes that modulate organic acid metabolism under salinity stress remain unidentified. Hu *et al.* [58] demonstrated that the vacuolar ATPase subunit MdVHA-B1 regulates salinity stress-induced malate accumulation in apple.

#### Antioxidant metabolism

Numerous studies have investigated antioxidant metabolism in fruit under salinity stress. In tomato, the intensity of salinity stress influences oxidative parameters (H_2_O_2_ and MDA levels), total ascorbate pool size, and the activities of antioxidant enzymes such as superoxide dismutase (SOD) and catalase (CAT). The activities and transcript levels of enzymes involved in the ascorbate–glutathione cycle, including ascorbate peroxidase (APX), monodehydroascorbate reductase (MDHAR), dehydroascorbate reductase (DHAR), and glutathione reductase (GR), also vary depending on the fruit developmental stage and stress intensity or duration [59]. Salinity stress affects ascorbate (AsA) and dehydroascorbate (DHA) contents, reflecting its impact on antioxidant metabolism. Salinity stress can also promote the expression of carotenoid biosynthesis genes, including *PSY1*, *PDS*, *ZDS*, and *LYCB*, correlating with increased lycopene, lutein, β-carotene, and violaxanthin contents [51, 60]. Salinity stress has also been reported to increase antioxidant activity in various fruit species. In goji berry (*Lycium barbarum* L.), severe salinity stress facilitates flavonoid glycosylation and carotenoid esterification [61]. In strawberry, moderate salinity increases antioxidant capacity, although the responses vary among cultivars. In the ‘Korona’ cultivar, salinity stress elevates SOD activity and increases glutathione, phenol, and anthocyanin contents. Meanwhile, in ‘Elsanta’, anthocyanin content decreases, and phenol levels remain similar. In fig, moderate salinity increases total phenol content by 5.6% [62].

#### Aroma and volatile compound metabolisms

Jin *et al.* [63] demonstrated that salinity stress significantly increases the number and concentration of volatile compounds, including alcohols, aldehydes, and esters. Salinity stress also markedly affects the levels of key volatiles such as hexanal, phenylethyl alcohol, and 6-methyl-5-hepten-2-one. In *Passiflora edulis*, lipoxygenase (LOX) plays a crucial role in volatile compound production. Huang *et al.* [64] showed that numerous *LOX* genes are responsive to abiotic stresses, including salinity stress, which is accompanied by an increase in total ester content.

### High temperature

#### Pigment metabolism

High temperature can strongly influence fruit coloration. Therefore, numerous studies have investigated the mechanisms underlying the modulation of pigment metabolism, particularly anthocyanin metabolism, in response to high-temperature stress. In tomato, elevated temperatures markedly reduce anthocyanin accumulation in the fruit peel [65]. In grape berries, the effects of high temperature on fruit coloration have been extensively studied, consistently showing that anthocyanin accumulation decreases significantly under heat stress [66–71]. In *Petunia hybrida*, the expression of *VviPrx31*, a gene encoding peroxidase, is strongly induced by high temperature. Overexpression of this gene decreases anthocyanin content in *P. hybrida* petals under heat stress, indicating that peroxidase activation inhibits anthocyanin accumulation at high temperatures [69]. More recently, Leng *et al.* [67] demonstrated that the TF VvMYB44-1 is involved in the inhibition of anthocyanin biosynthesis at high temperatures: *VvMYB44-1* expression is strongly induced by heat, and its overexpression suppresses anthocyanin accumulation. In banana, the inhibition of fruit coloration under high temperature may be related to sugar accumulation. High temperature accelerates fructose and glucose accumulation in the peel, and incubation of the peel with either sugar inhibits coloration [72].

MYB TFs play crucial roles in regulating plant pigmentation. Evidence indicates that these TFs are involved in the inhibition of apple fruit coloration by high temperature, as the expression of *MdMYB10*, a key gene in anthocyanin biosynthesis, is reduced in response to heat stress [73, 74]. Bu *et al.* [75] identified a LATERAL ORGAN BOUNDARIES (LOB) domain-containing gene, *MdLBD37*, whose expression is strongly upregulated by high temperature. Overexpression of this gene decreases anthocyanin accumulation and downregulates the expression of several anthocyanin biosynthesis genes, including *MdCHI*, *MdCHS*, *MdF3H*, *MdANS*, *MdDFR*, and *MdUFGT*, indicating that *MdLBD37* modulates fruit coloration under heat stress. Inhibition of anthocyanin accumulation by high temperature has also been reported in other fruit species, such as cherry, *Actinidia chinensis*, and cucumber [76–78]. ABA-insensitive 5 (ABI5), a pivotal TF in the ABA signaling pathway, has been implicated in high temperature–induced inhibition of fruit coloration in cucumber [76]. Silencing *ABI5* reduces high temperature–induced transcription of pheophytinase (*PPH*) and pheophorbide a oxygenase (*PAO*), two key genes in the chlorophyll catabolic pathway. Moreover, ABI5 interacts with MYB44, thereby alleviating the inhibitory effects of MYB44 on *PPH* and *PAO* transcription. Together, these results indicate that high temperature–induced fruit coloration results from enhanced chlorophyll degradation.

#### Sugar and organic acid metabolisms

Studies on the effects of high temperature on fruit quality have mainly focused on pigment metabolism and antioxidant activity, whereas investigations into sugar and organic acid metabolisms remain limited. Studies using SSR markers demonstrated that titratable acidity (TA) and total soluble solids (TSS) in tomato are higher under high temperatures than under ambient conditions [79]. Zheng *et al.* [80] reported that the combined treatment of high temperature and nitrogen fertilization influences sugar and acid metabolisms: appropriate nitrogen fertilizer application increases soluble sugar and organic acid contents in young tomato fruit under high temperature. Several genes involved in sugar metabolism and transport, such as those encoding sucrose-metabolizing enzymes (*CWINV2*, *HK2*, *SPS*, and *PK*) and sucrose transporters (*SUT1*, *SUT4*, and *SWEET*), are upregulated in response to the combined treatment. By contrast, in ‘Shiranuhi’ mandarin, total soluble sugar and acid contents decrease following high-temperature treatment [81]. Collectively, these findings indicate that the effects of high temperature on sugar and organic acid metabolisms vary depending on the fruit species and specific temperature regimes applied.

#### Antioxidant metabolism

AsA is well known as a potent antioxidant, and enzymes such as MDHAR and GR play crucial roles in AsA recycling. In tomato, high temperature significantly reduces MDHAR and GR activities [82]. Wang *et al.* [83] investigated the effects of various day/night temperature combinations (18/12°C, 25/12°C, 25/22°C, and 30/22°C) on antioxidant metabolism in strawberry. They found that increasing the night temperature from 12°C to 22°C while maintaining the day temperature at 25°C significantly elevates phenolic acid, flavonol, and anthocyanin contents. The highest day/night temperature combination (30°C/22°C) further enhances phenolic content and ROS-scavenging capacity. Zhu *et al.* [84] demonstrated that high temperature induces carotenoid production in banana pulp. The expression of several carotenoid biosynthesis genes, including *MaDXR1*, *MaPDS1*, *MaZDS1*, *MaLCYE*, *MaLCYB1.2*, *MaBCH*, and *MaSPL16*, is upregulated in response to high temperature. In addition, MaEIL9, an ethylene signaling component, regulates the transcription of *MaDXR1*, *MaPDS1*, *MaZDS1*, and *MaSPL16* by directly binding to their promoters. Expression of *MaEIL9* is strongly upregulated by high temperature, and its overexpression substantially enhances the expression of carotenoid biosynthesis genes, thereby increasing carotenoid content in tomato. Overall, these findings indicate that high temperature modulates carotenoid production via MaEIL9-mediated activation of carotenoid biosynthesis genes [84].

### Low temperature

A large number of studies have examined the effects of low temperature on fruit quality. However, most of them have focused on postharvest storage. In this section, we summarize the effects of low temperature on the development of fruit quality during the preharvest stage.

#### Pigment metabolism

Fruit pigmentation is regulated by both pigment accumulation and degreening caused by chlorophyll degradation. In apple, low temperature has been shown to induce anthocyanin biosynthesis [85, 86], although evidence also indicates that low temperature delays degreening [87]. By contrast, in lemon and ‘Satsuma’ mandarin, low temperature has been shown to trigger degreening [88, 89]. Although many studies have demonstrated that low temperature promotes anthocyanin accumulation [85, 90–94], Mao *et al.* [95] showed that low temperature inhibits anthocyanin accumulation in strawberry. A reasonable explanation for this discrepancy is that low temperature markedly delays fruit development and ripening, thus indirectly delaying anthocyanin accumulation. Therefore, when investigating the effects of low temperature on anthocyanin accumulation, it is important to distinguish whether these effects are direct or indirect. Xie *et al.* [96] demonstrated that a bHLH TF mediates low temperature–induced anthocyanin accumulation in apple. They isolated a bHLH TF designated as MdbHLH3 and found that *MdbHLH3* expression is strongly upregulated by low temperature. MdbHLH3 can bind to the promoters of *MdDFR* and *MdUFGT*, thereby promoting anthocyanin biosynthesis. These findings indicate that MdbHLH3 mediates low temperature-induced anthocyanin accumulation.

#### Sugar and organic acid metabolisms

Only a few studies have investigated the effects of low temperature on sugar and organic acid metabolisms. Alpha-amylase plays a vital role in starch metabolism, and in apple, the expression of an alpha-amylase gene is upregulated by low temperature [97], implying that starch degradation contributes to sugar metabolism under cold conditions. Low temperature–induced soluble sugar accumulation has also been reported in other fruit species, such as zucchini and kiwifruit [98, 99]. Li *et al.* [100] investigated the mechanism of low temperature-induced soluble sugar accumulation in apple and identified *MdTST1* and *MdTST2*, which encode tonoplast sugar transporters. Two low-temperature-responsive TFs, MdCBF1 and MdCBF2, have been shown to bind to the promoters of *MdTST1* and *MdTST2* and activate their expression. Overexpression of *MdCBF1* or *MdCBF2* increases the concentrations of glucose, fructose, and sucrose, while simultaneous silencing of *MdCBF1* and *MdCBF2* downregulates *MdTST1* and *MdTST2* expression and suppresses low temperature-induced sugar accumulation. These findings indicate that the MdCBF1/2–MdTST1/2 module mediates low temperature-induced sugar accumulation. Citrate is the primary organic acid in citrus fruit. Lin *et al.* [101] demonstrated that citrate content in *C. reticulata* Blanco cv. ‘Ponkan’ increases significantly in response to low temperature. This increase correlates with the upregulation of *CitPEPC*, *CitCS*, *CitGAD4*, and *CitAco3* by low temperature. These genes encode phosphoenolpyruvate carboxylase, citrate synthase, glutamate decarboxylase, and aconitase, respectively, enzymes involved in citrate metabolism. Accordingly, low temperature-induced citrate accumulation appears to result from complex regulation across multiple metabolic pathways.

#### Antioxidant metabolism

Only a limited number of studies have investigated the effects of low temperature on antioxidant metabolism. In strawberry, low temperature increases total polyphenol content [102]. Transcriptomic analysis revealed that several genes associated with phenylpropanoid biosynthesis are upregulated in response to low temperature, including those encoding shikimate O-hydroxycinnamoyl transferase, ferulate-5-hydroxylase, coniferyl-aldehyde dehydrogenase, and beta-glucosidase. Proanthocyanidins are potent antioxidants in fruits. Wang *et al.* [103] demonstrated that MYB TFs are involved in proanthocyanidin biosynthesis in apple. *MdMYBPA1* expression is induced by low temperature, while MdMYB9, MdMYB11, and MdMYB12 can bind to the *MdMYBPA1* promoter. Additionally, MdbHLH33 can directly bind to the low temperature-responsive (LTR) cis-element of the *MdMYBPA1* promoter. Overexpression of *MdMYBPA1* and *MdbHLH33* promotes anthocyanin production under low temperature. Similarly, in grape berries, several MYB-related genes, such as *VlMYBA1-3*, *VlMYBA1-2*, and *VlMYBA2*, are induced by low temperature [104]. Collectively, these observations indicate that MYB TFs play important roles in regulating antioxidant metabolism under low temperature. In addition to MYB TFs, WRKY TFs have been implicated in antioxidant metabolism. For example, in passion fruit (*Passiflora edulis*), *PeWRKY48* expression is strongly upregulated by low temperature, and *PeWRKY30* is associated with flavonoid accumulation, as suggested by metabolome–transcriptome co-expression analysis [105].

In summary, in the past decades, the physiological and molecular bases for the environmental modulation of fruit quality have been extensively investigated. To date, a large number of metabolic key genes and TFs have been found to be responsive to environmental factors (Table 1). These results have provided important information for further investigating the signaling mechanisms governing environmental modulation of fruit quality development. Comprehensive identification of the signaling modules between TFs and their target genes is especially important to unveil the signaling cascades for the environmental modulation of fruit quality development.

**Table 1:** The transcription factors, metabolic key genes and their target relation involved in environmental modulation of fruit quality

Environmental signals	Fruit quality	Species (English/Latin)	Transcript factors (Family)	Key enzymes of metabolims (Function)	Target relation	References
Water deficit	Pigmentation	Jujube/*Z. jujuba Mill.*		ZjANS (Anthocyanin synthase)		Wang *et al.* (2024)
		Tomato/*S. peruvianum*	SpAN2 (MYB)	SpDFR (Flavonoid biosynthetic)		Tapia *et al.* (2022)
		Grape/*V. vinifera L.*	VvAREB2 (ABA responsive), VvSBP8, VvSBP13, VvMYC1 and VvMYBA1		VvAREB2- Vv-miR156b- VvSBP8 and VvSBP13- VvMYC1 and VvMYBA1	Guo *et al.* (2024)
		Apple/*M. domestica*	MdERF38 and MdMYB1	MdDFR and MdUF3GT	MdERF38/MdMYB1-? MdDFR and MdUF3GT (?)	An *et al.* (2019)
	Content of sugar	Citrus/*C. reticulata Blanco*		CitSUS1, CitSUS2 and CitSUS3–5		Han *et al.* (2014)
				CwINV2/6 and SUS2/6		Khan *et al.* (2023)
				CsVPP-1, CsVPP-2 (vacuolar pyrophosphatase proton pump)		Hussain *et al.* (2020)
	Content of organic acid	Citrus/*C. reticulata Blanco*		CitIDH		Liu *et al.* (2007)
		Apple/*M. domestica*		MdPEPC4,		Wang *et al.* (2023)
	Content of antioxidant	Grape/*V. vinifera L.*		CHS2, CHS3, UFGT, F3H and F3’5’H		Castellarin *et al.* (2007)
		Strawberry/*Fragaria × ananassa*		PAL, 4CL, C4H2, DFR and FLS		Perin *et al.* (2019)
		Tomato/*S. lycopersicum*	SIMX1 (MYB)			Ewas *et al.* (2017)
	Content of aroma	Strawberry/*F. × ananassa*		PDC and AATs		Rodríguez-Arriaza *et al.* (2025)
Salinity	Pigmentation	Apple/*M. domestica*		GA2-oxidase (GA2ox)		Yan *et al.* (2024)
				MdUGT83L3		Li *et al.* (2022)
				MdHsp18.2b		Zhang *et al.* (2024)
	Content of Sugar and organic acid	Banana/*Musa acuminata*		SWEET (Sugar transporter)		Miao *et al.* (2017)
		Apple/*M. domestica*		V-ATPase MdVHA-B1		Hu *et al.* (2015)
	Content of antioxidant	Tomato/*S. lycopersicum*		SOD, CAT, DHAR, MDHAR, APX and GR		Murshed *et al.* (2013)
				PSY1, PDS, ZDS and LYCB		Leiva-Ampuero. *et al.* (2020)
	Content of aroma and volatile compounds	Passion fruit/*Passiflora edulis*		Lipoxygenase (LOX)		Huang *et al.* (2022)

(Continued)

**Table 1:** Continued

Environmental signals	Fruit quality	Species (English/Latin)	Transcript factors (Family)	Key enzymes of metabolims (Function)	Target relation	References
High temperature	Pigmentation	Grape/*V. vinifera L.*		VviPrx31 (Peroxidase)		Movahed *et al.* (2016)
			VvMYB44–1			Leng *et al.* (2025)
		Apple/*M. domestica*	MdMYB10			Lin-Wang *et al.* (2011) Rehman *et al.* (2017)
				MdLBD37		Bu *et al.* (2022)
		Cucumber/*Cucumis sativus L.*	ABI5 and MYB44	PPH and PAO	ABI5-MYB44-PPH/PAO	Liu *et al.* (2023)
	Content of sugar and organic acid	Tomato/*S. lycopersicum*		CWINV2, HK2, SPS, PK, SUT1, SUT4 and SWEETs		Zheng *et al.* (2023)
	Content of antioxidant	Banana/*M. acuminata*	MaEIL9	MaDXR1, MaPDS1, MaZDS1, MaLCYE, MaLCYB1.2, MaBCH and MaSPL16	MaEIL9-MaDXR1/MaPDS1/MaZDS/MaSPL16	Zhu *et al.* (2023)
Low temperature	Pigmentation	Apple/*M. domestica Borkh.*	bHLH	MdDFR and MdUFGT	bHLH- MdDFR/MdUFGT	Xie *et al.* (2012)
		Strawberry/*F. vesca*	FvMYB10	FvCHI	FvMYB10- FvCHI	Mao *et al.* (2022)
	Content of sugar and organic acid	Apple/*M. domestical*	MdCBF1 and MdCBF2	MdTST1/2 (Sugar Transporter)	MdCBF1/2- MdTST1/2	Li *et al.* (2024)
		Citrus/*C. reticulate* Blanco cv. Ponkan		CitPEPC, CitCSs, CitAco3 and CitGAD4		Lin *et al.* (2016)
	Content of antioxidant	Apple/*M. domestical*	MdMYB9/11/12, MdbHLH33 and MdMYBPA1		MdMYB9/11/12/MdbHLH33- MdMYBPA1	Wang *et al.* (2018)
		Grape/*V. labruscana*	VlMYBA1–3, VlMYBA1–2 and VlMYBA2			Azuma *et al.* (2012)
		Passion fruit/*Passiflora edulis*	PeWRKY48/30			Ma *et al.* (2024)

‘Target relation’ refers to that the transcription factors directly bind to the promoters of their target genes. Blank cells, no information available; ‘?’ denote ‘putative’

## Signaling components mediating fruit responses to environmental signals

Signal transduction underlying the environmental modulation of fruit quality traits involves three major biological processes: perception of environmental signals; cellular signaling relays; and the final signal output, i.e., the regulation of fruit quality-associated metabolisms. A wide range of signaling components are involved in these processes, among which phosphorylation modification catalyzed by protein kinases and phosphatases has been well established as the core mechanism. In addition to phosphorylation, ubiquitination and sumoylation are also essential to cellular signal transduction. Although these signaling components have been extensively reviewed, their specific roles in fruit trees, particularly in the context of fruit quality modulation, have not been systematically summarized. In the following section, we summarize the signaling components involved in stress responses in fruit trees, while the mechanisms by which these components modulate fruit quality development are discussed separately in the section on ‘Signaling cascades’. The related information about the signaling components is provided in Table 2.

**Table 2:** The signaling components involved in environmental modulation of fruit quality development

Family/subfamily	Environmental signals	Signaling components	Species (English/Latin)	References
Environmental sensors	Osmotic stress	AHK1	Arabidopsis/*A. thaliana*	Urao *et al*. (1999)
		DROOPY LEAF1 (DPY1)	Setaria/*Setaria italica*	Zhao *et al.* (2023)
	Thermal stress	Early flowering 3 (ELF3)	Arabidopsis/*A. thaliana*	Jung *et al.* (2020)
		THERMO-WITH ABA-RESPONSE 1 (TWA1)	Arabidopsis/*A. thaliana*	Bohn *et al.* (2024)
		FERONIA	Arabidopsis/*A. thaliana*	Liu *et al.* (2024)
MAPK	Cold, osmotic and salt stress	Multiple members of MAPK/MAPKK/MAPKKK	Banana/*M. acuminata*; Strawberry/*Fragaria × ananassa;* tomato/*S. lycopersicum*; Jujube/*Z. jujuba Mill*; water melon/*C. lanatus*	Wang *et al.* (2017); Zhou *et al.* (2017); Shi *et al.* (2025); Wang *et al.* (2025); Song *et al.* (2015)
	Cold	MKK2	Banana/*M. acuminata*	Gao *et al.* (2017)
	Cold	MAPK3	Strawberry/*Fragaria × ananassa*	Mao *et al.* (2022)
SnRK2s	dehydration	Multiple members of SnRK2	Sweet cherry/*P. avium*	Shen *et al.* (2017)
	Cold, osmotic and salt stress	Multiple members of SnRK2	Pepper/*C. annuum L.*	Wu *et al.* (2020)
CIPK	Salt stress	Multiple members of CIPK	Apple/*M. domestical*	Niu *et al.* (2018)
CDPK	Various abiotic stresses	Multiple members of CDPK	Strawberry/*Fragaria × ananassa*	Crizel *et al.* (2020); Llop-Tous, *et al.* (2002)
	Various abiotic stresses	Multiple members of CDPK	Peach/*Prunus persica‌*	Zhao *et al.* (2022)
	Various abiotic stresses	Multiple members of CDPK	Banana/*M. acuminata*	Li *et al.* (2019)
Protein Phosphatases	Drought, salt and ABA	Multiple members of PP2C	Strawberry/*Fragaria × ananassa*	Haider *et al.* (2019)
	Abiotic stresses (cold, heat, and drought)	Multiple members of PP2C	litchi /*Litchi chinensis Sonn*	Yang *et al.* (2025)
	Abiotic stresses	Multiple members of PP2C	Tomato/*S. lycopersicum*	Zhang *et al.* (2018)
E3-ligase	High temperature	Ubiquitin E3 Ligase	Banana/*M. acuminata*	Wei *et al.* (2020); (2023)
	Cold stress	SINA Ubiquitin Ligase MaSINA1	Banana/*M. acuminata*	Fan *et al.* (2017)
	Cold stress	E3 ligase MdSIZ1	Apple/*M. domestical*	Zhou *et al.* (2017)

Blank cells, no information available; MAPK, mitogen activated protein kinase; SnRK2, SNF1-related protein kinase 2; CIPK, CBL-interacting protein kinase; CDPK, calcium depdendent-protein kinase; PP2C, protein phosphotase 2C.

### Environmental sensors

The key step in signal transduction initiated by environmental factors is the perception of the environmental signals, a process believed to be mediated by environmental sensors. In recent years, the identification of environmental sensors that perceive water availability, salinity, and temperature has attracted considerable attention from plant scientists. To date, several putative environmental sensors have been reported in plants. More than two decades ago, Urao *et al.* [106] demonstrated that a transmembrane hybrid-type histidine kinase (AHK1) in *Arabidopsis* functions as an osmosensor. A subsequent study [107] suggested that AHK1 also regulates stomal density, thereby influencing plant responses to low water potential. Recently, DROOPY LEAF1 (*DPY1*), which encodes a leucine-rich repeat receptor-like kinase (LRR-RLK), has been identified as another osmosensor [108, 109]. Several proteins have also been proposed to function as temperature sensors, including photoreceptors and early flowering 3 [110–112]. Importantly, a recent study by Bohn *et al.* [113] demonstrated that THERMO-WITH ABA-RESPONSE 1 (TWA1) functions as a temperature sensor. *TWA1* was identified by screening ABA signaling–associated mutants for thermosensitivity. It encodes a predicted 130 kDa intrinsically disordered protein. While the function of the protein is unknown, it contains two potential ethylene-responsive-element-binding-factor-associated amphiphilic repression (EAR) motifs. In addition to the abovementioned thermosensors, plasmalemma-localized protein kinases such as FERONIA have also been suggested to function as temperature sensors [9]. Although limited information is available regarding the roles of environmental sensors in fruit quality development, several studies have reported that FERONIA participates in the regulation of fruit ripening [114–116]. A comprehensive understanding of the roles of environmental sensors in fruit development is therefore crucial for elucidating the mechanisms underlying environmental modulation of fruit quality.

### MAPK cascades

MAPK cascades are key signaling modules that govern plant development and responses to abiotic and biotic stresses [117]. A typical MAPK cascade consists of MAPK, MAPK kinase (MAPKK), and MAPKK kinase (MAPKKK). While the functions of MAPK cascades in plant development and stress responses have been extensively reviewed [117–120], their roles in fruit responses to environmental signals have not been summarized. Evidence indicates that MAPK cascades play essential roles in regulating fruit development in response to environmental signals. Wang *et al.* [121] identified 10 *MAPKK* and 77 *MAPKKK* genes in the banana genome. In the *MAPKK* gene family, 50.0%, 60.0%, and 80.0% of genes are upregulated in response to cold, osmotic, and salinity stresses, respectively, while in the *MAPKKK* gene family, 43.4%, 48.7%, and 59.2% genes are upregulated under the same conditions. Additionally, MKK2, a member of the MAPKK family, has been found to be phosphorylated in response to cold stress [122]. Similarly, Zhou *et al.* [123] identified 12 *FvMAPK*, 7 *FvMAPKK*, 73 *FvMAPKKK*, and one *FvMAPKKKK* gene in the strawberry genome, most of which are significantly upregulated under cold and salt stresses. The responses of MAPK cascades to abiotic stresses have also been reported in several other fruit species, including tomato, jujube, *Prunus mume*, and watermelon [124–127]. Collectively, these findings suggest that the MAPK cascades play crucial roles in environmental stress-induced signaling in fruits.

### SnRK2s and CBL-CIPKs

The sucrose non-fermenting protein kinase 1 (SNF1)-related protein kinase (SnRK) family contains a conserved serine/threonine protein kinase domain at the N-terminal and can be classified into three subgroups: SnRK1, SnRK2, and SnRK3. The latter two subgroups are specific to plants and are known as stress-activated protein kinases (SAPKs) and CIPKs, respectively. SnRKs play central roles in plant stress signaling [11, 128–130]. Shen *et al.* [131] identified six *PacSnRK2* members in the sweet cherry genome, all of which are upregulated in response to dehydration stress. The pepper (*Capsicum annuum* L.) genome contains nine putative *SnRK2* genes, most of which are responsive to various abiotic stresses [132]. Similarly, *SnRK2* expression in cucumber is responsive to abiotic stresses [133]. Several studies have also reported the involvement of CIPKs in fruit responses to salinity stress. For example, the apple genome contains 34 *MdCIPK* genes, of which 21 are upregulated under salinity stress [134].

### CDPKs

Ca^2+^ signaling serves as a crucial second messenger in almost all plant responses to abiotic stresses [135]. As central Ca^2+^ sensor proteins, CDPKs are essential mediators of these responses [136, 137]. Multiple studies have shown that CDPKs are involved in fruit responses to environmental signals. For example, nine *CDPK* genes have been identified in strawberry, and nearly all are responsive to various abiotic stresses [138]. Similarly, Llop-Tous *et al.* [139] reported that a *CDPK* gene in strawberry is regulated by cold stress. Zhao *et al.* [140] identified 17 *PpCDPK* genes in the peach genome, most of which show significant expression changes in response to abiotic stresses. Li *et al.* [141] identified 44 *CDPK* genes in banana, many of which respond to abiotic stresses. These studies suggest that CDPKs play important roles in mediating fruit responses to environmental stresses.

### Protein phosphatases

Protein phosphorylation plays a crucial role in plant stress responses, with protein phosphatase 2C (PP2C) proteins acting as key regulators in various signaling pathways [142]. In addition to PP2Cs, protein tyrosine phosphatases (PTPs) have been increasingly recognized as important in plant growth and abiotic stress responses [143]. Evidence indicates that protein phosphatases are involved in stress responses in fruits. A genome-wide analysis [144] revealed that the strawberry genome contains 62 PP2C-encoding genes categorized into 12 subgroups, many of which are highly regulated in response to drought, salinity, and ABA stresses. Most of the genes are upregulated, with a small portion downregulated. Similarly, the litchi (*Litchi chinensis* Sonn.) genome contains 68 *PP2C* genes categorized into 13 groups, most of which are differentially expressed under cold, heat, and drought stresses [145]. Expression changes in *PP2C* genes under abiotic stresses have also been reported in tomato [146]. These studies demonstrate that PP2Cs are involved in stress responses in fruit trees. However, to date, no information is available regarding the role of PTPs in fruit responses to abiotic stress.

### E3 ligases

Ubiquitination and sumoylation are crucial regulatory mechanisms that govern the selective degradation and stabilization of regulatory proteins through the ubiquitin–proteasome system (UPS), in which E3 ligases play a central role. These ligases interact with target proteins in a substrate-specific manner, thereby regulating protein degradation, activity, and cellular localization [147, 148]. Thus, ubiquitination and sumoylation constitute an essential part of cellular signal transduction. It has been increasingly recognized that these processes are involved in diverse biological processes, including plant responses to environmental stresses [149, 150]. Increasing evidence suggests that E3 ligases play important roles in regulating fruit development under abiotic stress. For example, in banana, E3 ligases are involved in high temperature–induced green ripening [151, 152]. Moreover, the SINA ubiquitin ligase MaSINA1 regulates cold stress responses by controlling the stability of MaICE1 [153]. Zhou *et al.* [123] reported that the small ubiquitin-like modifier E3 ligase MdSIZ1 promotes anthocyanin accumulation in apple by sumoylating MdMYB1 under low-temperature conditions.

## Signaling cascades governing environmental modulation of fruit quality development

The signaling cascades governing the environmental modulation of fruit quality can be divided into two major mechanisms. The first involves direct regulation of key enzymes associated with fruit quality–related metabolism, while the second involves regulation of hormonal signal production, which subsequently governs fruit ripening and quality development.

### Signaling cascades mediating fruit quality–associated metabolisms

#### Pigment metabolism

Cullin–RING finger ligases (CRLs) constitute the largest family of ubiquitin ligases in eukaryotic cells. Cullin acts as a scaffold that interacts with specific adaptors and diverse substrates in a combinatorial manner [154]. Evidence indicates that CRLs participate in the environmental modulation of pigment metabolism. In tomato, two high-pigment mutants (*hp1* and *hp2*) were identified based on elevated levels of fruit pigments. *HP1* and *HP2* encode DAMAGED DNA BINDING PROTEIN 1 (DDB1) and DE-ETIOLATED 1 (DET1), respectively. SlDDB1, SlDET1, and SlCUL4 form a CRL4 complex that targets proteins for degradation by the 26S proteasome [155]. Tang *et al.* [156] demonstrated that the CUL4–DDB1–DET1 E3 complex targets the GOLDEN 2-LIKE TF (GLK2) for degradation. GLK2 plays a critical role in chloroplast development, thereby influencing fruit pigmentation [156, 157]. In purple tomato fruits, anthocyanin biosynthesis is largely regulated by *HY5*, whose expression levels are controlled by E3 ligase COP1–mediated destabilization. Recently, Menconi *et al.* [65] reported that high temperatures trigger HY5 degradation under light conditions through its nuclear relocation and interaction with DET1 and COP1, demonstrating that the COP1–DET1–HY5 module acts as a crucial switch for high temperature–induced repression of anthocyanin synthesis. In pear (*Pyrus* spp.), the expression of *PpHY5L* is inhibited by HEAT SHOCK FACTOR B2A (PpHsfB2a) [158]. Moreover, the stability of PpHsfB2a is regulated by the RING-type E3 ubiquitin ligase PpATL52, leading to PpHsfB2a protein accumulation at high temperatures. Thus, the PpATL52–PpHsfB2a–PpHY5L cascade modulates anthocyanin biosynthesis in response to high temperatures. In banana, high temperatures (> 24°C) result in green ripening due to inhibited chlorophyll degradation. NON-YELLOW COLORING 1 (*MaNYC1*) has been identified as a regulator of chlorophyll degradation, and its protein levels decrease in response to high temperature. The RING-type E3 ligase NYC1-interacting protein 1 (MaNIP1) interacts with and ubiquitinates MaNYC1, leading to its proteasomal degradation, indicating that the MaNIP1–MaNYC1 module mediates temperature-induced green ripening in banana [159].

As summarized above, MYB TFs play crucial roles in regulating fruit quality development. Increasing evidence suggests that environmental modulation of fruit quality traits by MYB TFs also involves regulation through E3-ligase complexes. In red-skinned apples, *MdMYB1* regulates anthocyanin biosynthesis. The small ubiquitin-like modifier E3 ligase MdSIZ1 directly sumoylates MdMYB1 under moderately low temperatures, leading to MdMYB1 degradation. The transcriptional level of *MdSIZ1* is strongly induced by low temperature, and *MdSIZ1* overexpression promotes anthocyanin accumulation, indicating that the MdSIZ1–MdMYB1 module modulates color formation in response to low temperature [160]. Recently, Jiang *et al.* [161] reported that *MdMYB2* is also involved in the modulation of anthocyanin biosynthesis under low temperature. *MdMYB2* expression is induced by low temperature, and importantly, MdMYB2 can directly bind to the MBS motif in the *MdSIZ1* promoter to activate its expression. Expression of both *MdMYB2* and *MdSIZ1* enhances cold tolerance in plants. Thus, the MdMYB2–MdSIZ1–MdMYB1 cascade mediates low temperature–induced anthocyanin biosynthesis. In banana, high temperature suppresses peel yellowing, leading to green ripening. In this process, MaMYB60 plays a critical role in regulating five chlorophyll catabolic genes (*CCG* genes). Wei *et al.* [152] demonstrated that the RING-type E3 ligase MaBAH1 targets MaMYB60 for proteasomal degradation during green ripening, thus attenuating MaMYB60-induced expression of *CCG* genes and chlorophyll degradation under high-temperature stress. Collectively, these studies demonstrate that RING finger ligases are essential regulators of fruit pigmentation in response to temperature stress.

Protein kinases play crucial roles in environmental stress signaling [162–164], suggesting their involvement in the environmental modulation of fruit pigmentation. However, information on this aspect remains limited. Nevertheless, there is evidence that protein kinases participate in the environmental modulation of fruit pigmentation. In strawberry, low temperature inhibits anthocyanin accumulation via MAPK signaling, which acts as a negative regulator of anthocyanin biosynthesis. Low temperature activates FvMAPK3 activity, which subsequently attenuates fruit pigmentation [95]. On one hand, FvMAPK3 phosphorylates chalcone synthase 1 (FvCHS1), promoting its proteasome-mediated degradation; on the other hand, FvMAPK3 is activated by MAPK kinase 4 (FvMKK4) and SnRK2.6 (FvSnRK2.6) through direct phosphorylation. Thus, the FvMKK4/FvSnRK2.6–FvMAPK3–FvMYB10/FvCHS1 cascade mediates low temperature–inhibited pigmentation in strawberry.

#### Sugar and organic acid metabolisms

Salinity influences malate accumulation in apple, and the V-ATPase subunit MdVHA-B1 serves as a regulator of this process. In *Arabidopsis*, SALT OVERLY SENSITIVE2 (*SOS2*), which encodes a serine/threonine protein kinase, mediates salinity stress signaling. Similarly, MdSOS2L1 directly phosphorylates MdVHA-B1, thereby modulating salinity-induced malate accumulation [58]. Protein S-acyltransferases (PATs) catalyze the S-acylation of their target proteins. In apple, the expression of the PAT-encoding gene *MdPAT16* is induced by salinity stress and significantly promotes soluble sugar accumulation. MdPAT16 catalyzes the S-acylation of MdCBL1, a calcium sensor, and improves its stability. Subsequently, MdCBL1 regulates the activity of MdCIPK13, which phosphorylates MdSUT2.2, thereby modulating sucrose accumulation. Collectively, this MdPAT16–MdCBL1–MdCIPK13–MdSUT2.2 cascade mediates salinity-modulated sugar accumulation in apple [165].

#### Cuticle wax accumulation

As discussed above, in apple, the SUMO E3 ligase MdSIZ1 regulates the stability of MdMYB1, thereby modulating low temperature–induced anthocyanin biosynthesis [160]. Recently, Zhang *et al.* [166] reported that MdSIZ1 is also involved in the environmental modulation of wax accumulation and cuticle permeability in apple. They found that MdMYB30 modulates wax accumulation through direct regulation of β-ketoacyl-CoA synthase 1 (*MdKCS1*), which encodes a key enzyme in the wax biosynthesis pathway. Because *MdSIZ1* expression is regulated by MdMYB2 in response to low-temperature stress, it is reasonable to propose that the MdMYB2–MdSIZ1–MdMYB30 cascade mediates low temperature–induced wax accumulation. Overall, current evidence indicates that RING-type E3 ligases play essential roles in regulating diverse fruit quality traits in response to environmental signals.

**Figure 1:**
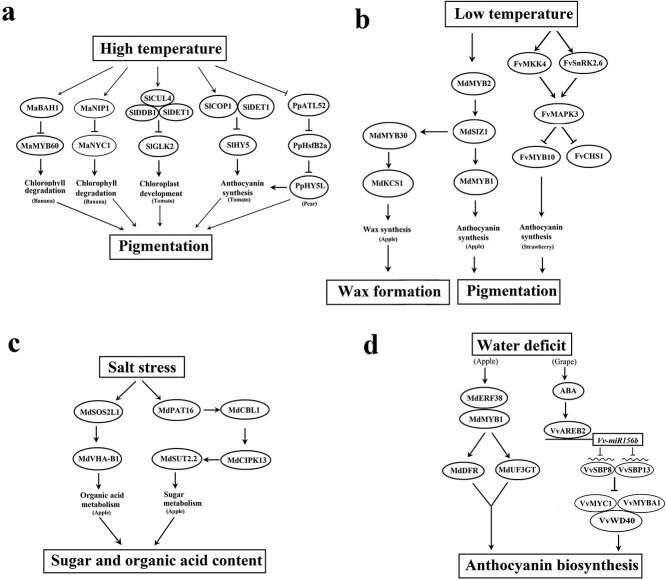
The signaling cascades that mediate environmental modulation of fruit quality related metabolisms. In a, RING-type E3 ligase: MaBAH1, MaNIP1, PpATL52; E3 ligase complex: SlCUL4, SlDDB1 and SlDET1; SlCOP1 and SlDET1; Transcription factors: MaMYB60, MaNYC1, SlGLK2, SlHY5, PpHY5L; HEAT SHOCK FACTOR: PpHsfB2a. In b, Transcription factors: MdMYB2, MdMYB30, MdMYB1; FvMYB10. MdSIZ1, a E3 ligase; MdKCS1, β-KETOACYL-COA SYNTHASE; FvCHS1, a chalcone synthase‌; FvMKK4, a MAPK kinase; FvMAPK3, a mitogen-activated protein kinase; FvSnRK2.6, a SNF1-related protein kinases 2. In c, MdSOS2L1, a protein kinase; MdVHA-B1, a V-ATPase subunit; MdPAT16, a protein S-acyltransferase; MdSUT2.2, a sucrose transporter; MdCBL1, a calcium sensor; MdCIPK13, CBL-interacting protein kinase. In d, Transcription factors: MdERF38, MdMYB1, VvAREB2, VvMYC1, VvMYBA1, VvWD40; Key enzymes: MdDRF, MdUF3GT. →，activation; ⊥, repression; interconnected ellipses, complex.

In summary (Fig. 1), a number of signaling cascades have been found to be implicated in the environmental modulation of fruit quality development. Notably, the signaling cascades vary depending on plant species, leading to our lack of an integrated picture of the signaling network for the environmental modulation of fruit quality trait. Because of this, it is important to use the model fruits, such as tomato and strawberry, to demonstrate whether the cascades found in different plant species occur in the model plants.

### Signaling cascades mediating hormonal signal production

Fruit ripening and quality development are two closely associated processes. Environmental modulation of fruit quality development can occur either through the direct regulation of fruit quality–associated metabolisms or through the regulation of hormonal signal production, thereby influencing fruit quality traits. Ethylene is a pivotal hormone governing climacteric fruit ripening. Several studies have provided valuable insights into the signaling mechanisms underlying ethylene production and transduction in response to environmental stresses. In tomato, for example, the MAPK LeMAPK4 has been suggested to participate in the regulation of ethylene production under cold stress [167]. Similarly, as recently demonstrated [168], SlMAPK3 mediates cold stress–induced ethylene production. The transcription level of *SlMAPK3* is markedly increased under cold treatment, and *SlMAPK3* overexpression upregulates genes encoding key enzymes in the ethylene biosynthesis pathway, including *SlACS2* and *SlACS4*. In apple, Jia *et al.* [169] demonstrated explored the roles of SnRK2 protein kinases in ETH biosynthesis related to fruit ripening and osmoregulation. The researchers identified two MdSnRK2-I members, MdSnRK2.4 and MdSnRK2.9, that were significantly upregulated during ripening or following mannitol treatment. MdSnRK2-I phosphorylated the TFs MdHB1 and MdHB2 to enhance their protein stability and transcriptional activity on MdACO1. MdSnRK2-I also interacted with MdACS1 and increased its protein stability through two phosphorylation sites. As thus, the MdSnRK2-I function to meditate osmotic stress-induced ETH production via through the regulation of both MdACO1 and MdACS1 metabolic pathways [169].

ABA is a pivotal hormone regulating non-climacteric fruit ripening. It is well established that ABA biosynthesis can be induced by various environmental stresses, particularly drought stress [170, 171]. Therefore, it is reasonable to propose that environmental modulation of non-climacteric fruit ripening and quality development involves the regulation of ABA biosynthesis. However, mechanistic information on this aspect remains limited. A recent study by Jia *et al.* [172] citation number reported that a MADS-box SEPALLATA 4 (SEP4) subfamily TF, FaCMB1, acts as a negative regulator of strawberry ripening. FaCMB1 represses the expression of *FaASR*, a stress-responsive TF, by directly binding to its promoter. FaASR, in turn, inhibits the transcriptional activity of *FaCYP707A4*, a key ABA 8′-hydroxylase enzyme involved in ABA catabolism. Furthermore, FaCMB1 can be phosphorylated by the kinase FaSTPK, and this phosphorylation regulates the binding affinity of FaCMB1 to the *FaASR* promoter. Thus, a signaling cascade that regulates ABA biosynthesis, i.e., FaSTPK–FaCMB1–FaASR–FaCYP707A4–ABA catabolism, has been elucidated. Although *FaASR* is a stress-responsive TF, it remains unclear how this signaling cascade mediates environmental modulation of ABA levels, thereby influencing strawberry fruit quality development [172].

**Figure 2:**
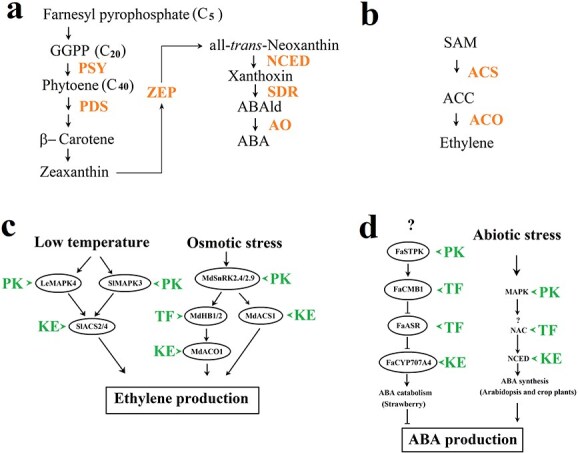
The signaling cascades that mediate environmental modulation of hormonal signal production in fruits. a and b, ABA and ethylene biosynthesis pathways. In c, LeMAPK3/4, mitogen-activated protein kinases; MdSnRK2.4/2.9; SNF1-related protein kinase 2. MdHB1/2, Homeobox transcription factors; MdACS1, an ACC synthase; MdACO1, an ACC oxidase. In d, FaSTPK, a protein kinase; FaCMB1, a MADS-box SEPALLATA transcription factor; FaASR, a stress responsive transcription factor; FaCYP707A4, an enzyme catalyzing ABA catabolism. MAPK, mitogen activated protein kinase; NAC, transcription factor. Abbreviations: KE, key enzyme; PK, protein kinase; PSY, phytoene synthase; PDS, phytoene desaturase; ZEP, zeaxanthin epoxidase; NCED, 9-cis-epoxycarotenoid dioxygenase; SDR, short-chain dehydrogenase/reductase; AO, aldehyde oxidase; ACS, ACC synthase. →, activation; ⊥, repression.

In summary (Fig. 2), the signaling cascades mediating environmental modulation of hormonal signal transduction have been increasingly identified. The MAPK and SnRK2-associated signaling pathways have been well established to play crucial roles in the modulation of ETH signal production. Unfortunately, less information is available about the signaling mechanisms for the environmental modulation of ABA signal production. The related information from *Arabidopsis* and some crop plants are valuable for further studies on the signaling mechanism controlling ABA signal production. Some signaling components and TFs, such as MAPKs, SnRK2s, NACs, and HBs should be paid particular attentions for deeply decoding the signaling network implicated in the environmental modulation of ABA signal production in fruits.

## Conclusion and perspectives

Fruit quality trait is largely determined by environmental factors. This review aims to summarize the knowledge on the signaling cascades in the environmental modulation of fruit quality trait. Reversible phosphorylation catalyzed by protein kinases and phosphatases represents a major mechanism controlling cellular signal transduction, whereas protein modification catalyzed by E3 ligases regulates the stability and subcellular localization of signaling proteins, therefore constituting an essential component of cellular signaling processes. As the end-event of the signaling output, regulation of the TFs functions to control the activities of key enzymes in fruit quality-associated metabolisms. In the past decades, while a large number of TFs together with their target genes have been identified, the signaling cascades upstream of the TFs is still poorly understood. To date, a comprehensive and explicit picture depicting the signaling mechanism for the environmental modulation of fruit quality formation has been lacking in a specific fruit species. Accordingly, further deciphering the signaling cascades upstream of the TFs would still be a major task for the mechanistic studies on fruit quality development. To facilitate comprehension and guide future research endeavors, we propose several perspectives on the signaling cascades involved in the modulation of fruit quality development.

Although numerous TFs that regulate fruit quality development, along with their target genes, have been characterized, there are still many knowledge gaps from the initial perception of environmental stimuli to the regulation of the TFs. Specifically, information about the signaling components, modules and cascades upstream of the TFs is still not enough to depict a systemic and explicit picture for the environmental modulation of fruit quality trait. Therefore, comprehensively deciphering the signaling cascades upstream of the TFs should still be a major aim in the basic studies on fruit quality development.

### Environmental sensors

Environmental modulation of fruit quality relies on environmental sensors that perceive environmental signals and trigger corresponding signaling cascades to regulate fruit quality–associated metabolisms. To date, several putative environmental sensors have been identified in *Arabidopsis*, including the osmosensors AHK1 and DPY1, and the thermosensors ELF3, CLK, TWA1, and FERONIA. However, studies on environmental sensors have primarily focused on their roles in plant stress tolerance, and currently, no information is available regarding their involvement in the environmental modulation of fruit quality development. Stable transgenic techniques, such as CRISPR/Cas9 and gene overexpression, are essential for functional studies of environmental sensors. Given that reliable and efficient transgenic systems have been well established in model fruit species such as tomato and strawberry, the use of these models will greatly accelerate research progress. Exploring the roles of environmental sensors in fruit quality development is therefore of particular significance for deepening our understanding of the molecular mechanisms underlying fruit quality development in response to environmental signals.

### Reversible phosphorylation

Reversible phosphorylation catalyzed by protein kinases and phosphatases is a major mechanism of cellular signal transduction and is thus expected to play crucial roles in the environmental modulation of fruit quality development. Protein kinases and phosphatases together constitute a superfamily comprising several hundred members in common plant species. Although some reports have indicated that protein kinases are involved in the environmental modulation of fruit quality in certain plant species, the number of identified members remains insufficient to provide a comprehensive understanding of how reversible phosphorylation regulates fruit quality–associated metabolisms under various environmental conditions. SnRKs and MAPK signaling modules (i.e., MAPKKK–MAPKK–MAPK modules) are widely recognized as key regulators that enable plants to cope with environmental stresses. Comprehensive screening of SnRKs, MAPKKK/MAPKK/MAPK family members, and other protein kinases and phosphatases involved in the environmental modulation of fruit quality should therefore be prioritized in future studies. The use of fruit transient expression techniques in combination with multiomic analyses will greatly facilitate the efficient identification and characterization of the protein kinases and phosphatases associated with fruit quality modulation.

### E3 ligases

Protein modification catalyzed by E3 ligases is an essential component of cellular signal transduction due to its roles in regulating the behavior of signaling proteins and TFs. Although several studies have reported the involvement of E3 ligases in the environmental modulation of fruit quality development, most of these have focused on the regulation of fruit pigmentation in response to temperature stress. Increasing evidence suggests that E3 ligases play important roles in the modification of MYB TFs, as discussed earlier. Nevertheless, the number of MYB proteins identified as E3-ligase substrates remains limited. Given the established roles of MYBs in regulating fruit quality–associated metabolisms, elucidating the roles of E3 ligases in MYB modification will advance our understanding of the mechanisms governing the environmental modulation of fruit quality development. Additionally, because many WRKY TFs have been identified as regulators of fruit quality development, comprehensive screening for WRKY members that can be modified by E3 ligases is particularly important for advancing mechanistic understanding in this area.

### TFs and their target genes

The identification and characterization of TFs involved in fruit quality development and ripening have been major research focuses in horticultural science. To date, numerous TFs have been demonstrated to play important roles in regulating fruit development and ripening. However, information regarding their roles in the environmental modulation of fruit quality development remains limited. A comprehensive investigation into the expression patterns of TFs in response to environmental signals will facilitate the efficient identification of TFs involved in this modulation. Among these, the MYB and WRKY families are most frequently reported to participate in fruit quality regulation. Therefore, a systematic analysis of their expression responses to various environmental signals will help to identify specific members that mediate environmental modulation of fruit quality development. Increasing evidence suggests that individual TFs can target multiple genes, thereby synergistically orchestrating diverse metabolic pathways. Exploring the mechanisms by which individual TFs regulate multiple genes will enhance understanding of how different fruit quality parameters are interconnected. Combining multiple biological techniques, such as fruit transient expression assays, transcriptomic analysis, bioinformatic identification of cis-elements, and electrophoretic mobility shift assays (EMSAs), is essential for the efficient identification of TF target genes.

### Plant hormones

Plant hormones act as internal signals that regulate fruit ripening and quality development. Hormone levels, determined by the balance between biosynthesis and catabolism, can be modulated in response to environmental signals. Consequently, alterations in environmental conditions are expected to modulate fruit ripening and quality development through their effects on hormone biosynthesis and degradation. The key enzymes involved in the biosynthesis and catabolism of major plant hormones have been identified and characterized. Further identification of the TFs regulating these enzymes will represent a breakthrough in elucidating the signaling cascades that govern hormonal biosynthesis and catabolism. For example, homeobox (HB) TFs have been demonstrated to regulate ethylene biosynthesis [169], while NAC TFs have been shown to regulate ABA biosynthesis [173–175]. However, it remains unclear whether these TFs can respond to environmental signals and thereby modulate fruit quality development. Comprehensive screening for HB and NAC TFs that not only regulate ethylene and ABA biosynthesis but also respond to environmental signals will provide deeper insights into the mechanisms governing the environmental modulation of fruit quality development.

### Glossary of key terms

Environmental modulation, environmental sensor, fruit quality formation, high temperature, hormonal signal production, low temperature, physiological and molecular bases, reversible phosphorylation, signaling cascade; target genes, water deficit.
